# Risk contagion of COVID-19 to oil prices: A Markov switching GARCH and PCA approach

**DOI:** 10.1371/journal.pone.0312718

**Published:** 2024-12-05

**Authors:** Nida Siddiqui, Haslifah Mohamad Hasim

**Affiliations:** 1 Department of Actuarial Mathematics and Statistics, Heriot-Watt University, Dubai, United Arab Emirates; 2 Department of Mathematics, University of Sharjah, Sharjah, United Arab Emirates; University of Malta / University of Turin / IPAG Business School / London School of Economics, ITALY

## Abstract

The COVID-19 pandemic and its impact on crude oil prices created additional risks throughout the financial industry. To contribute to the ongoing debates, this paper empirically examined the risk contagion of COVID-19 to oil prices by incorporating a Markov-Switching GARCH (MS-GARCH) framework and the multivariate GARCH time series model, BEKK-GARCH model. The study examines data collected between 27 January 2020 and 31 December 2020. Further, we used principal component analysis (PCA) to find principal factors explaining the overall variability of the global economic indicators that contribute to the risk. Finally, to support the risk transmission effects between COVID-19 and oil prices, we conducted regression analysis, while controlling for the factors extracted from the PCA method.

## Section 1: Introduction

Crude oil prices play a crucial role in world economics, and the more volatile crude oil prices become, the more uncertainty they create, leading to economic instability for both oil-exporting and oil-importing countries [1]. Crude oil prices do not work in isolation. Crude oil price fluctuations impact economic growth, stock prices, bond markets, stock returns and national security [2, 3]. Apart from the influence of demand and supply, crude oil prices are also subject to the effects of exchange rates, market speculation, and geopolitical scenarios. [4] gives different theoretical reasons for how crude oil price shocks impact different macroeconomic variables. Hence, understanding the volatility of oil prices and their correlation with other commodities, financial instruments, and geopolitical situations remains an important domain of research. According to a study by [5], shocks in the oil price volatility demonstrate spillover effects. Hence, changes in their volatility can expose producers, consumers, investors and portfolio managers to risk, affecting investment portfolios, government policies, and world economies at large.

Oil prices and hence their volatility are not merely determined by economic variables but also as a result of unexpected events. The COVID-19 pandemic was no exception. COVID-19 has had an enormous impact on the oil and financial markets across the globe. The uncertainty and risk perception created by the pandemic caused volatility in financial markets and commodity prices [6].

Therefore, this paper explores the dynamics between the COVID-19 pandemic and global oil prices by studying the risk contagion between the two. The empirical investigations are focused on Dubai crude, which is one of the main three benchmarks in oil pricing along with West Texas Intermediate (WTI) and Brent, covering daily oil prices data for the period from 27 January 2020 until 31 December 2020. In this context, we first performed the Markov-switching generalized autoregressive conditional heteroskedasticity (GARCH) model to examine whether the crude oil prices result in a regime shift by the COVID-19 pandemic. Furthermore, we investigated the correlation between oil prices and COVID-19 mortality and recovery rates, in addition to analysing the global macroeconomic factors. To this end, we used the multivariate GARCH time series model, BEKK-GARCH model to explore the risk contagion effect between COVID-19 and oil prices.

This paper contributes to the literature on the impact of COVID-19 to crude oil prices. While several recent studies, e.g., [7, 8] have examined the oil price volatility during the COVID-19 pandemic, and [9] explores the dynamics of this volatility and explains the effects of these shocks induced by oil demand and supply, this paper is unique in its contribution to the literature as it explores the direct impact of the COVID-19 indicators to the crude oil prices. Moreover, we further explored the impact of these indicators on oil prices while controlling for the economic risk dispersion factor and the economic instability factor extracted from the principal component analysis (PCA) of the global macroeconomic variables.

The rest of this paper is structured as follows: Section 2: Literature Review provides a comprehensive review of the related studies, Section 3: Theoretical Framework presents the theoretical framework and methodology used in this study, Section 4: Empirical Analysis exhibits the empirical results, and Section 5: Conclusion summarises the main conclusions drawn from this study.

## Section 2: Literature review

A large number of studies have been carried out over recent years to investigate the impact of COVID-19 on oil and financial markets. Most of these studies have shown that the COVID-19 pandemic has intensified the volatility of oil and financial markets, which created a significant negative impact on risk contagion across the markets.

During this period, the huge price volatility in the energy market dominated by crude oil caused an increase in the risk contagion effect. Many of these recent studies concentrate on the profound understanding of the risk contagion effect between oil and other financial markets; for examples [10] investigates the dynamics of heterogeneous frequency of risk spillovers across the major financial markets (i.e., the stock market, commodity market, bond market, and foreign exchange market), [11] explores the multidimensional risk spillovers among crude oil and the US and Chinese stock markets, and [8] examines the risk contagion between the oil and Gulf Cooperation Council (GCC) stock markets.

The methodology employed in some existing studies examining the risk contagion during the COVID-19 pandemic period has involved the utilization of the vector autoregressions (VAR) family model, for examples [12, 13] use the time-varying parameter VAR (TVP-VAR) approach of [14, 15] utilises a high-dimensional and time-varying factor-augmented VAR model (HD-TVP-FAVAR).

A different set of models employed to analyse the risks associated with conditional volatility is the generalized autoregressive conditional heteroskedasticity (GARCH) model, along with its multivariate extensions, which are utilized to assess the risk contagion effects between crude oil prices and various financial markets. A study by [16] computes the intraday volatility estimates by employing the multiplicative component GARCH (MCS-GARCH) model introduced by [17]. This particular model decomposes the volatility of high-frequency returns into multiplicative components, allowing for simpler estimation and interpretation. They then employed spillover index approaches of [18] to approximate risk contagion between stocks and major commodity markets, including oil, during the COVID-19 pandemic.

The two most widely used models of conditional covariances and correlations in the class of multivariate GARCH models are the Baba-Engle-Kraft-Kroner GARCH (BEKK-GARCH) and dynamic conditional correlation GARCH (DCC-GARCH) models [19]. Within the domain of DCC-GARCH models, [9] studies the volatility spillovers and co-movements among energy-related stocks during the outbreak of COVID-19 using DCC-FIGARCH, a dynamic conditional correlation (DCC) model extended to a multivariate fractionally integrated generalized ARCH (FIGARCH) framework, which accounts for long memory and time-varying correlations. A research carried out by [20] examines risk transmission between oil and precious metal markets using the DCC-GARCH model. They found that the COVID-19 pandemic has strengthened the transfer of volatility from oil to precious metals. In the study conducted by [21], a DCC model was applied to the Fractionally Integrated GARCH (FIGARCH) model to investigate the transmission mechanism of volatility shocks and the contagion effects between stock and commodity markets. Further, [22] investigates the impacts of infectious disease pandemics such as COVID-19 on the long-term volatility and correlation between crude oil and gold markets using the DCC mixed data sampling (DCC-MIDAS) approach originally proposed by [23].

However, [19] concluded in their summary that, from a theoretical perspective, the BEKK-GARCH model is the optimal model for estimating conditional covariances (and thereby also conditional correlations). Furthermore, [24] also discovered that BEKK-GARCH models better captured asymmetric volatility spillover compared to DCC-GARCH models. The main advantage of the BEKK-GARCH model is that it ensures positive definiteness of the variance-covariance matrix [25]. [26] found that BEKK-GARCH offers a more flexible parameterisation with positive definite conditional covariance matrices, making it suitable for modeling high volatility shocks in multivariate time series data compared to DCC-GARCH. Studies such as [27] have also suggested that the BEKK-GARCH framework is better than other GARCH models when assessing risk contagion. It is also worth noting that many studies, e.g., [28] have suggested that the BEKK-GARCH model is superior to other GARCH models in terms of forecasting ability.

With respect to the impact of the COVID-19 pandemic on financial markets, [29] uses the BEKK-GARCH model to analyse the risk contagion through spillover prior to and during the COVID-19 pandemic in cross-market BRIC countries, i.e., Brazil, Russia, India, and China, which are identified as rising economic powers. A study by [30] employs a combination of the Markov-switching GARCH model and the BEKK-GARCH model to examine the transmission of volatility from energy markets, such as crude oil, to financial markets across different regimes during the COVID-19 pandemic period. In another interesting study, [31] uses the multivariate BEKK-GARCH model to measure the volatility spillover effect from the COVID-19 news, proxied by daily death rate and recovered rate, to the stock market.

Given the advantages of the BEKK-GARCH model in detecting risk contagion effects, we employed this model as one of the primary methodologies in our study.

Although the conditional volatility GARCH models perform well with linear time series, they are unable to represent many nonlinear dynamic patterns such as asymmetry and amplitude [32]. The Markov switching model, also known as the regime-switching model, is one of the most popular nonlinear time series models in the literature. [33] demonstrates the superior performance of the Markov switching conditional volatility models in cases where volatility experiences regime changes. Therefore, we incorporated the Markov-Switching GARCH models into the multivariate BEKK-GARCH framework to examine whether the COVID-19 pandemic resulted in a regime shift. Since our study involved the assessment of various macroeconomic variables, we also incorporated principal component analysis (PCA), a widely utilised method for dimensionality reduction. Moreover, we utilised the method of [32] to evaluate the direct impact of COVID-19 indicators on crude oil prices.

## Section 3: Theoretical framework

### Markov switching GARCH (MS-GARCH) models

GARCH models have been extensively used to capture the time-varying volatility in economic time series. However, the volatility predictions by GARCH-type models may fail to capture true variation in volatility in case of regime changes in the volatility dynamics [33]. An approach to address this issue is to allow the parameters of the GARCH model to vary through a Markov process. Therefore, to determine whether COVID-19 caused a regime shift in the Dubai crude oil prices, we employed the Markov-switching GARCH (MS-GARCH) model.

Let *y*_*t*_ be the variable of interest, the log returns at time *t*. To simplify the exposition, we assumed that *y*_*t*_ has zero mean and do not exhibit serial correlation. We denoted the information set up to time *t* − 1 by $I_{t-1}$, i.e., $I_{t-1}=\{y_{t-i},i>0\}$.

The MS-GARCH specification of [33] is as follows:

$$\begin{array}{c} y_{t}|\left(s_{t}=k,I_{t-1}\right)∼D\left(0,h_{k,t},\xi_{k}\right), \end{array}$$ (1)

where $D(0,h_{k,t},\xi_{k})$ is a continuous (conditional) distribution with zero mean, *h*_*k*,*t*_ is the time-varying variance, and additional shape parameters (e.g., asymmetry and kurtosis) gathered in the vector *ξ*_*k*_. For *t* = 1, we initialised the regime probabilities and the conditional variances at the unconditional level. The integer-valued stochastic variable *s*_*t*_, defined on the discrete space {1, …, *K*}, characterises the MS-GARCH model.

We further assumed that *s*_*t*_ evolves according to an unobserved first-order ergodic homogeneous Markov chain with *K* × *K* transition probability matrix **P** such that $\text{P}\equiv {\left\{p_{i,j}\right\}}_{i,j=1}^{K}$. Here, *p*_*i*,*j*_ ≡ *P*[*s*_*t*_ = *j*|*s*_*t*−1_ = *i*] is the probability of transition from *s*_*t*−1_ = *i* to *s*_*t*_ = *j*.

Given the parametrisation of D(·), we have $E\left[y_{t}^{2}|s_{t}=k,I_{t-1}\right]=h_{k,t}$. In this model, the conditional variances *h*_*k*,*t*_ for *k* = 1, …., *K* are assumed to follow *K*-*separate* GARCH-type processes which evolve in parallel. The conditional variance dynamics is such that depending upon *s*_*t*_ = *k*, *h*_*k*,*t*_ is available as a function of the past observation *y*_*t*−1_, past variance *h*_*k*,*t*−1_ and the additional regime-dependent parameters *θ*_*k*_:

$$\begin{array}{c} h_{k,t}\equiv h\left(y_{t-1},h_{k,t-1},\theta_{k}\right), \end{array}$$ (2)

where *h*(⋅) is a *I*_*t*−1_ measurable function, which defines the filter for the conditional variance and also ensures that it is positive. Furthermore, it is assumed that *h*_*k*,1_(*k* = 1, …, *K*) are set equal to the unconditional variance in regime *k*. Depending on the form of *h*(⋅), different scedastic specifications are obtained. For instance, if

$$\begin{array}{c} h_{k,t}\equiv \omega_{k}+\alpha_{k}y_{t-1}^{2}+\beta_{k}h_{k,t-1}, \end{array}$$ (3)

with *ω*_*k*_ > 0, *α*_*k*_ > 0, *β*_*k*_ > 0 such that the covariance-stationarity is ensured by *α*_*k*_ + *β*_*k*_ < 1, we obtained the MS-GARCH(1,1) model. Thus, we have *θ*_*k*_ = (*ω*_*k*_, *α*_*k*_, *β*_*k*_)^*T*^.

Alternative specifications of *h*(⋅) can be seamlessly integrated into the model, such as incorporating the asymmetric Exponential GARCH (EGARCH) scedastic specification [34] to effectively capture the asymmetric responses of time-varying variance to shocks and leverage effects. This can be expressed as:

$$\begin{array}{c} \text{ln}\left(h_{k,t}\right)\equiv \omega_{k}+\alpha_{k}(|\eta_{k,t-1}|-E[|\eta_{k,t-1}|])+\gamma_{k}\eta_{k,t-1}+\beta_{k}\;\text{ln}\left(h_{k,t-1}\right), \end{array}$$ (4)

for *k* = 1, …., *K*, where the expectation *E*[|*η*_*k*,*t*−1_|] is taken with respect to the distribution conditional on regime *k*. Covariance-stationarity in each regime is obtained by requiring that *β*_*k*_ < 1.

Another specification of *h*(⋅) can be

$$\begin{array}{c} h_{k,t}\equiv \omega_{k}+\left(\alpha_{k}+\gamma_{k}I\left\{y_{t-1}<0\right\}y_{t-1}^{2}\right)+\beta_{k}h_{k,t-1}, \end{array}$$ (5)

which gives the Glosten-Jagannathan-Runkle GARCH (GJR-GARCH) model of [35]. This model enables to capture the asymmetry in the conditional variance process, i.e., that good news and bad news have different effects on the conditional volatility. In the model, I is the indicator function with *k* reflecting the regimes, and *γ*_*k*_ captures the asymmetry component. In this case, we have *θ*_*k*_ = (*ω*_*k*_, *α*_*k*_, *γ*_*k*_, *β*_*k*_)^*T*^.

Covariance-stationarity of the variance process conditional on the Markovian state is achieved by imposing *α*_*k*_ + *β*_*k*_ + *κ*_*k*_*γ*_*k*_ < 1, where $\kappa_{k}\equiv P\left[y_{t}<0|s_{t}=k,I_{t-1}\right]$.

We considered two different choices for D(.), the standard normal (N) and Student-t (S) distributions. In this study, we used a total of 18 GARCH model specifications as a result of the combinations based on

- number of regimes, *K* such that *K* ∈ (1, 2),
- conditional variance specifications: GARCH, EGARCH, and GJR-GARCH, and
- the choice of the conditional distribution $D(0,h_{k,t},\xi_{k})$.

GARCH parameters can be estimated using maximum likelihood procedure [36], and the best fitting GARCH models are chosen based on Akaike Information Criterion (AIC) and Bayesian Information Criterion (BIC).

### Principal Component Analysis (PCA)

This paper uses the principal component analysis (PCA) as the dimension reduction method to find parsimonious principal factors that explain the overall variability of macroeconomic variables. To this end, we performed the eigenvalue decomposition on the covariance matrix. More specifically, let **Z** denote a random vector of standardised macroeconomic indicators, *PC*_*i*_ as *i*^*th*^ principal component of **Z**, and *e*′_*i*_ as factor loadings.

The principal components are as follows:

$$\begin{array}{c} PC=\left(\begin{array}{c} PC_{1}\\ PC_{2}\\ \vdots \\ .\\ PC_{p} \end{array}\right) \end{array}=\left(\begin{array}{c} e_{1}^{\prime }Z\\ e_{2}^{\prime }Z\\ \vdots \\ .\\ e_{p}^{\prime }Z \end{array}\right)=\text{e}\cdot \text{Z}.$$ (6)

Then the random vector **Z** is written as

$$\begin{array}{c} \text{Z}=(\begin{array}{ccc} e_{11} & \cdots & e_{p1}\\ \vdots & \ddots & \vdots \\ e_{1p} & \cdots & e_{pp} \end{array})(\begin{array}{c} PC_{1}\\ \vdots \\ .\\ PC_{p} \end{array})={\text{e}}^{\text{T}}\cdot \text{PC}. \end{array}$$ (7)

We used the parsimonious principal factors to estimate the original normalized values $\tilde{\text{Z}}$, which is specified as

$$\begin{array}{c} \tilde{\text{Z}}={\text{e}}_{\text{R}}^{\text{T}}.{\text{PC}}_{\text{R}}. \end{array}$$ (8)

Based on the reduced form from Eq (7), we found the two most influential principal factors, namely the systematic risk (*SR*) and the economic interdependence factor (*EIF*). The detailed results of this will be discussed in Section 4: Empirical Analysis on macroeconomic variables and PCA.

### Risk contagion: Multivariate BEKK-GARCH

To examine the direct impact of COVID-19 indicators on the Dubai crude oil, we employed the multivariate BEKK-GARCH model with the parametrization proposed by [25]. This model deals with the interaction between time-varying variances and covariances, making it particularly useful for capturing the risk contagion effect, also often referred to as volatility spillover effects, between variances.

In this model, **H**_*t*_ is the conditional covariance matrix of *k*-dimensional random vector *ϵ*_*t*_,

$$\begin{array}{c} H_{t}=CC^{\prime }+A^{\prime }\epsilon_{t-1}\epsilon_{t-1}^{\prime }A+B^{\prime }H_{t-1}B. \end{array}$$ (9)

Here, **C** denotes a lower triangular matrix, **A** is a matrix of ARCH terms, whereas **B** is a matrix of GARCH terms. However, the parametrization of Eq (9) is restricted to symmetric effects. We therefore employed the extension developed by [37] to capture the asymmetry which is specified as follows:

$$\begin{array}{c} H_{t}=CC^{\prime }+A^{\prime }\epsilon_{t-1}\epsilon_{t-1}^{\prime }A+B^{\prime }H_{t-1}B+D^{\prime }\nu_{t-1}\nu_{t-1}^{\prime }D, \end{array}$$ (10)

where the matrix **D** measures asymmetries, and *ν*_*t*_ is the adjustment for asymmetry that is defined as *ν*_*t*_ = *ϵ*_*t*_ if *ϵ*_*t*_ < 0 (i.e., negative shocks), and *ν*_*t*_ = 0 otherwise.

The unrestricted BEKK model based on Eq (10) in multivariate form is given by:

$$\begin{array}{cc} \left(\begin{array}{ccc} h_{11,t} & h_{12,t} & h_{13,t}\\ h_{21,t} & h_{22,t} & h_{23,t}\\ h_{31,t} & h_{32,t} & h_{33,t} \end{array}\right) & =\left(\begin{array}{ccc} c_{11} & 0 & 0\\ c_{21} & c_{22} & 0\\ c_{31} & c_{32} & c_{33} \end{array}\right){\left(\begin{array}{ccc} c_{11} & 0 & 0\\ c_{21} & c_{22} & 0\\ c_{31} & c_{32} & c_{33} \end{array}\right)}^{\prime }\\ & +{\left(\begin{array}{ccc} a_{11} & a_{12} & a_{13}\\ a_{21} & a_{22} & a_{23}\\ a_{31} & a_{32} & a_{33} \end{array}\right)}^{\prime }\left(\begin{array}{ccc} \epsilon_{1,t-1}^{2} & \epsilon_{1,t-1}\epsilon_{2,t-1} & \epsilon_{1,t-1}\epsilon_{3,t-1}\\ \epsilon_{1,t-1}\epsilon_{2,t-1} & \epsilon_{2,t-1}^{2} & \epsilon_{2,t-1}\epsilon_{3,t-1}\\ \epsilon_{1,t-1}\epsilon_{3,t-1} & \epsilon_{2,t-1}\epsilon_{3,t-1} & \epsilon_{3,t-1}^{2} \end{array}\right)\\ & \;\;\;\;\;\;{\left(\begin{array}{ccc} \epsilon_{1,t-1}^{2} & \epsilon_{1,t-1}\epsilon_{2,t-1} & \epsilon_{1,t-1}\epsilon_{3,t-1}\\ \epsilon_{1,t-1}\epsilon_{2,t-1} & \epsilon_{2,t-1}^{2} & \epsilon_{2,t-1}\epsilon_{3,t-1}\\ \epsilon_{1,t-1}\epsilon_{3,t-1} & \epsilon_{2,t-1}\epsilon_{3,t-1} & \epsilon_{3,t-1}^{2} \end{array}\right)}^{\prime }\left(\begin{array}{ccc} a_{11} & a_{12} & a_{13}\\ a_{21} & a_{22} & a_{23}\\ a_{31} & a_{32} & a_{33} \end{array}\right)\\ & +{\left(\begin{array}{ccc} b_{11} & b_{12} & b_{13}\\ b_{21} & b_{22} & b_{23}\\ b_{31} & b_{32} & b_{33} \end{array}\right)}^{\prime }\left(\begin{array}{ccc} h_{11,t-1} & h_{12,t-1} & h_{13,t-1}\\ h_{21,t-1} & h_{22,t-1} & h_{23,t-1}\\ h_{31,t-1} & h_{32,t-1} & h_{33,t-1} \end{array}\right)\left(\begin{array}{ccc} b_{11} & b_{12} & b_{13}\\ b_{21} & b_{22} & b_{23}\\ b_{31} & b_{32} & b_{33} \end{array}\right)\\ & +{\left(\begin{array}{ccc} d_{11} & d_{12} & d_{13}\\ d_{21} & d_{22} & d_{23}\\ d_{31} & d_{32} & d_{33} \end{array}\right)}^{\prime }\left(\begin{array}{c} v_{1,t}\\ v_{2,t}\\ v_{3,t} \end{array}\right){\left(\begin{array}{c} v_{1,t}\\ v_{2,t}\\ v_{3,t} \end{array}\right)}^{\prime }\left(\begin{array}{ccc} d_{11} & d_{12} & d_{13}\\ d_{21} & d_{22} & d_{23}\\ d_{31} & d_{32} & d_{33} \end{array}\right) \end{array}$$ (11)

The diagonal elements of **A** indicate the specific ARCH effect of the fluctuations in the crude oil prices, COVID-19 recovery, and COVID-19 death rate on the crude oil prices, reflecting the short-term persistence of shocks, whereas the off-diagonal elements represent the cross-shock spillovers. The matrix **B** contains the GARCH coefficients of the model, where the diagonal elements are the own GARCH effect, representing the long-run volatility persistence of these shocks on the crude oil prices, while the off-diagonal elements represent the risk contagion effects of these COVID-19 indicators on the crude oil prices. If any coefficient in matrix **D** is positive and statistically significant, it indicates the presence of an asymmetric effect where bad news leads to greater volatility compared to good news. Conversely, if the coefficient is not significant, it suggests that the impact of bad news may be similar to that of good news. A negative coefficient implies an opposite effect, potentially resulting in decreased volatility or even stability in the crude oil market.

Expanding matrix **H**_*t*_ in Eq (11) allows us to observe more clearly how volatility is influenced. As an example, for

$$\begin{array}{cc} h_{11,t} & =c_{11}^{2}+(a_{11}\epsilon_{1,t-1}+a_{21}\epsilon_{2,t-1}+a_{31}\epsilon_{3,t-1}{)}^{2}+b_{11}^{2}h_{11,t-1}\\ & +b_{21}^{2}h_{22,t-1}+b_{31}^{2}h_{33,t-1}+2b_{11}b_{21}h_{12,t-1}+2b_{11}b_{31}h_{13,t-1}+2b_{21}b_{31}h_{23,t-1}\\ & +(d_{11}v_{1,t-1}+d_{21}v_{2,t-1}+d_{31}v_{3,t-1}{)}^{2}. \end{array}$$ (12)

The results of this analysis will be discussed in Section 4: Empirical Analysis.

### Regression equation and parameters

To investigate the combined impact of macroeconomic variables and COVID-19 indicators, we formulated our regression equation as follows:

$$\begin{array}{c} COPR_{t}=\lambda +\phi SR_{t}+\psi EIF_{t}+\tau Death_{t}+\delta Recovery_{t}+e_{t}. \end{array}$$ (13)

The *COPR*_*t*_ in Eq (13) represents the crude oil prices risk at any given time *t*, calculated as the standard deviation in the returns of crude oil prices over a 20-day rolling window. *SR*_*t*_ and *EIF*_*t*_ are the systematic risk and economic interdependence factors, respectively. These are extracted as the two main components from the principal component analysis (PCA) of the macroeconomic variables. *Death*_*t*_ is the standardised global death rate due to COVID-19, and *Recovery*_*t*_ is the standardised global recovery rate of COVID-19 patients.

### Data set

The dataset on crude oil prices consists of the daily closing price of DAS-1M, which is a crude oil blend from the United Arab Emirates (UAE), obtained from Refinitiv Datastream, covering the period between 27 January 2020 until 31 December 2020. The daily log returns for the oil prices are calculated as $R_{t}=100*log\left(\;\frac{P_{t}}{P_{t-1}}\;\right)$. The mean is close to zero, with a standard deviation of 4.013 and a kurtosis of 17, suggesting non-normality in oil returns. We proceeded with conducting tests to assess the presence of heteroskedasticity in the data. The Ljung-Box and Box-pierce tests gave significant *χ*^2^ values for all lags. The results are shown in Table 1.

**Table 1:** Descriptive statistics of oil returns.

Descriptive statistics of oil returns			
Mean	-0.057	Skewness	-2.1
Minimum	-32.2	Kurtosis	17
Maximum	12.5	Jarque Bera Test	3072*
Std. Dev.	4.013	Ljung-Box test(all lags)	p<0.05

Note:

* refers to statistical significance of 1%.

**Fig 1:**
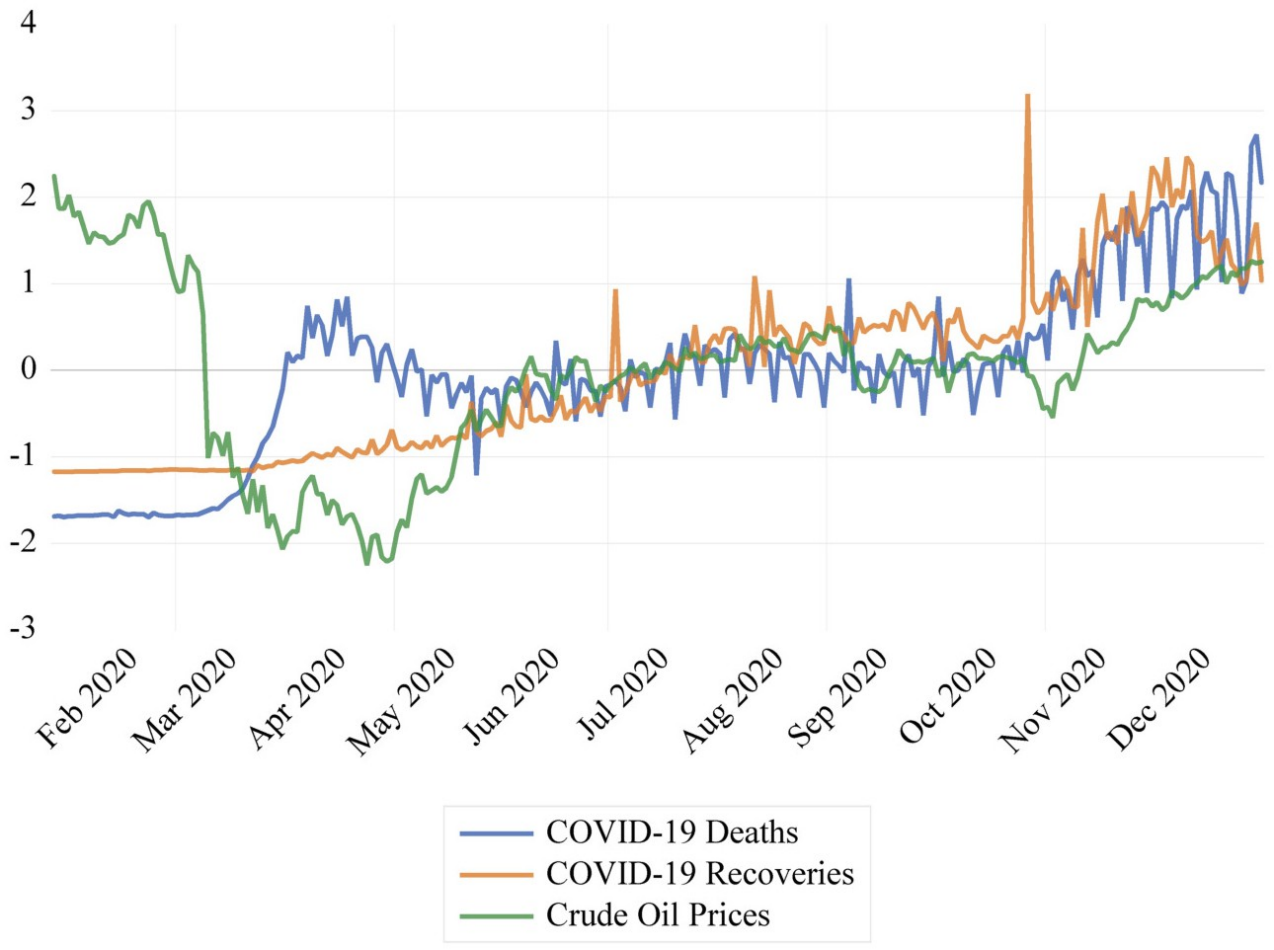
Standardised Crude oil prices, COVID-19 recoveries, and COVID-19 deaths.

Similarly, the data on global COVID-19 deaths and recoveries was collected from the Johns Hopkins Coronavirus Resource Centre (https://coronavirus.jhu.edu/data) for the same period. The length of the data set was determined by the initial availability of death and recovery rates data, as well as the rollout of the first COVID-19 vaccines after the approval of the World Health Organisation (WHO) in the final week of December 2020. The data for all three variables was recorded over a period of five weekdays, excluding weekends, and subsequently converted into a time series. Fig 1 shows the evolution of the standardized variables over the selected period. For more details, see S1 Table in the Supporting Information document.

**Fig 2:**
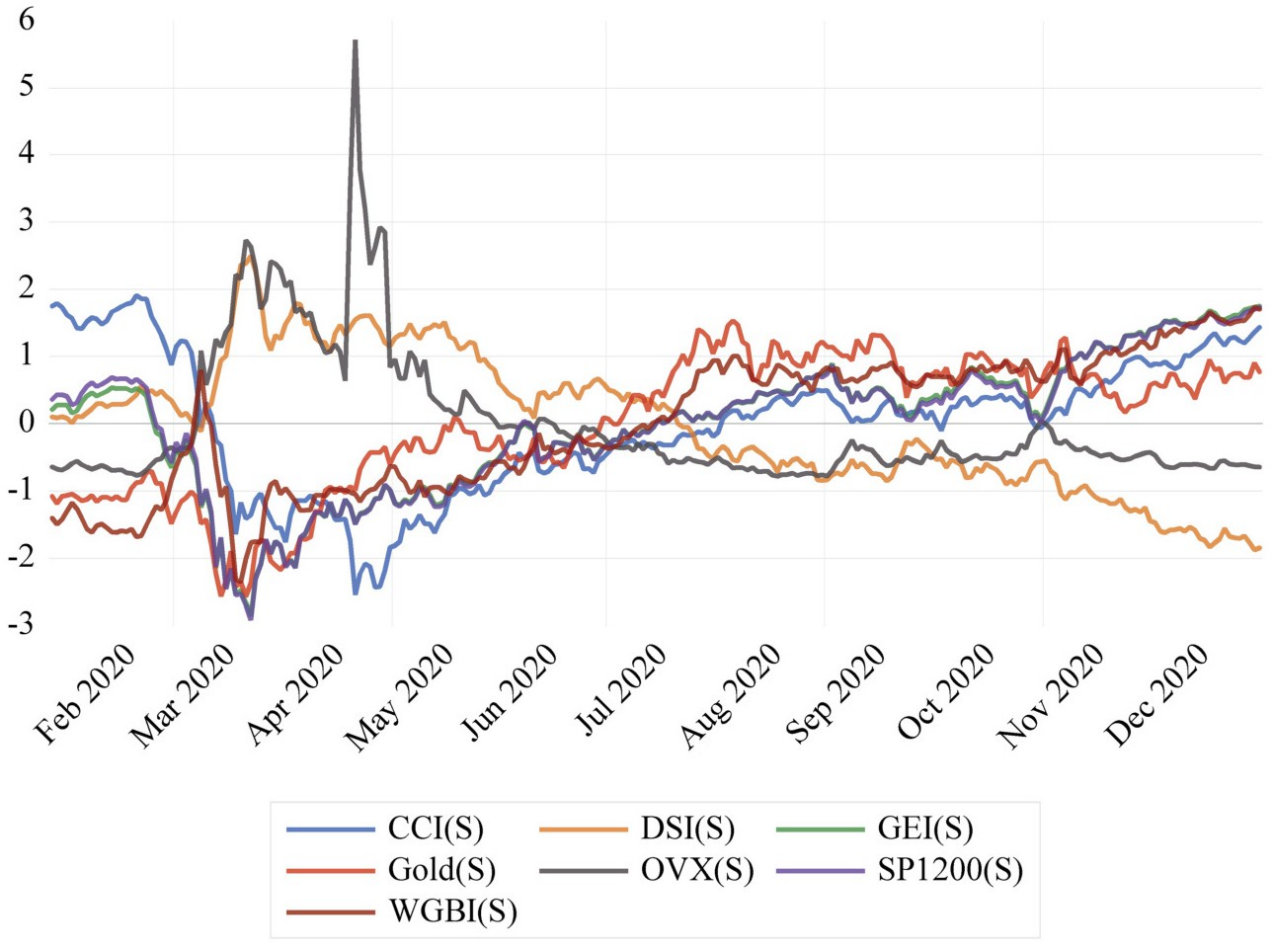
Standardised macroeconomic variables: CCI-Core Commodity Index, DSI-Dollar Spot Index, GEI-MSCI Global Equity Index, Gold: daily gold prices, OVX CBOE Crude Oil Volatility Index, SP1200-S&P Global 1200, and WGBI-FTSE World Government Bond Index.

The global macroeconomic indicators include the Cboe Crude Oil ETF Volatility Index, the MSCI Global Equity Index, the FTSE World Government Bond Index (FTSE WGBI), the Dollar spot index, CoreCommodity Index(CRB), the daily gold prices, and S&P Global 1200. These indicators were recorded for the same period, and standardiZed, and their time series is illustrated in Fig 2. We observed a chaotic pattern in the behavior of macroeconomic variables as the number of deaths increased.

## Section 4: Empirical analysis

**Fig 3:**
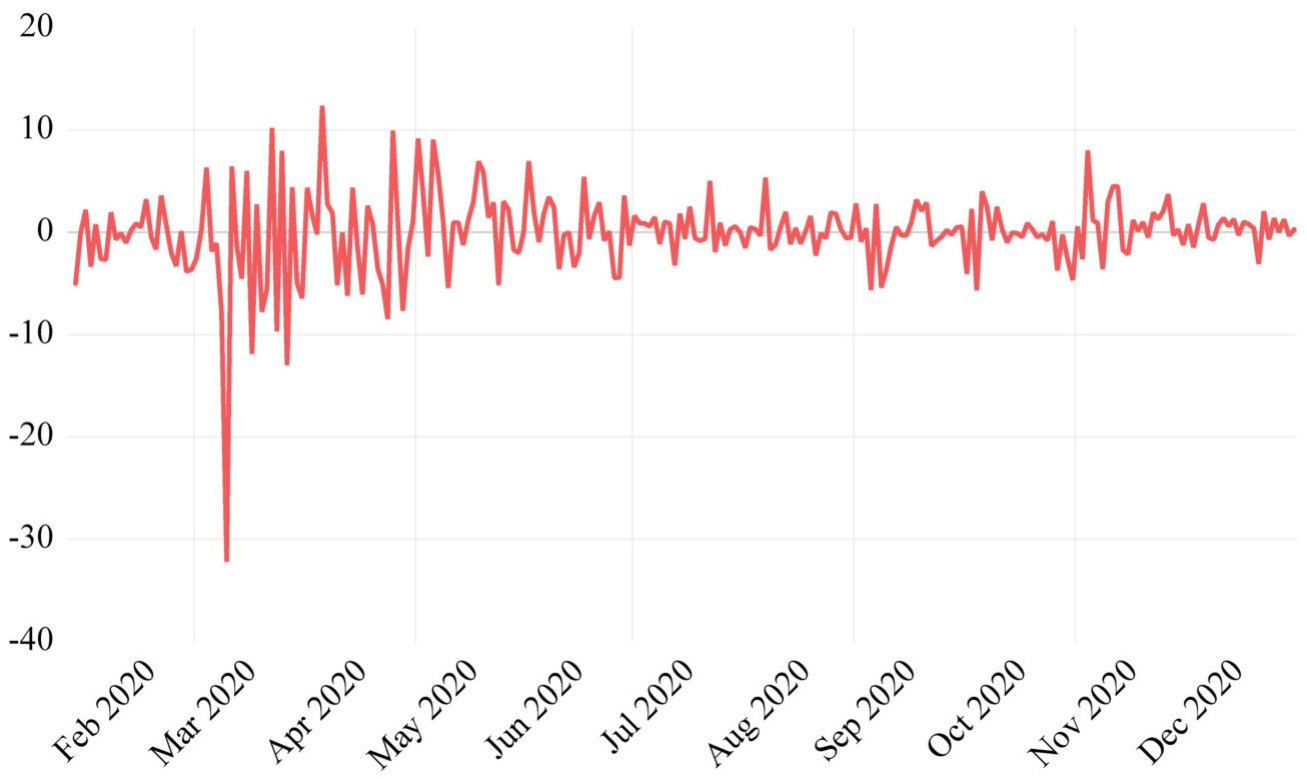
Crude oil prices returns.

Our analysis commenced with the computation of log returns for crude oil prices. Fig 3 shows the log-returns of the crude oil. The augmented Dickey-Fuller (ADF) test confirms stationarity, whereas volatility clustering is confirmed by conducting the Lagrange Multiplier (ARCH-LM) test for heteroskedasticity.

### MS-GARCH regime switching

The first step of the analysis focuses on determining the presence of a regime shift in crude oil prices during the COVID-19 period. To achieve this objective, we employed the MS-GARCH models. The findings confirm a regime shift occurring from early February to 30 June 2020.

**Fig 4:**
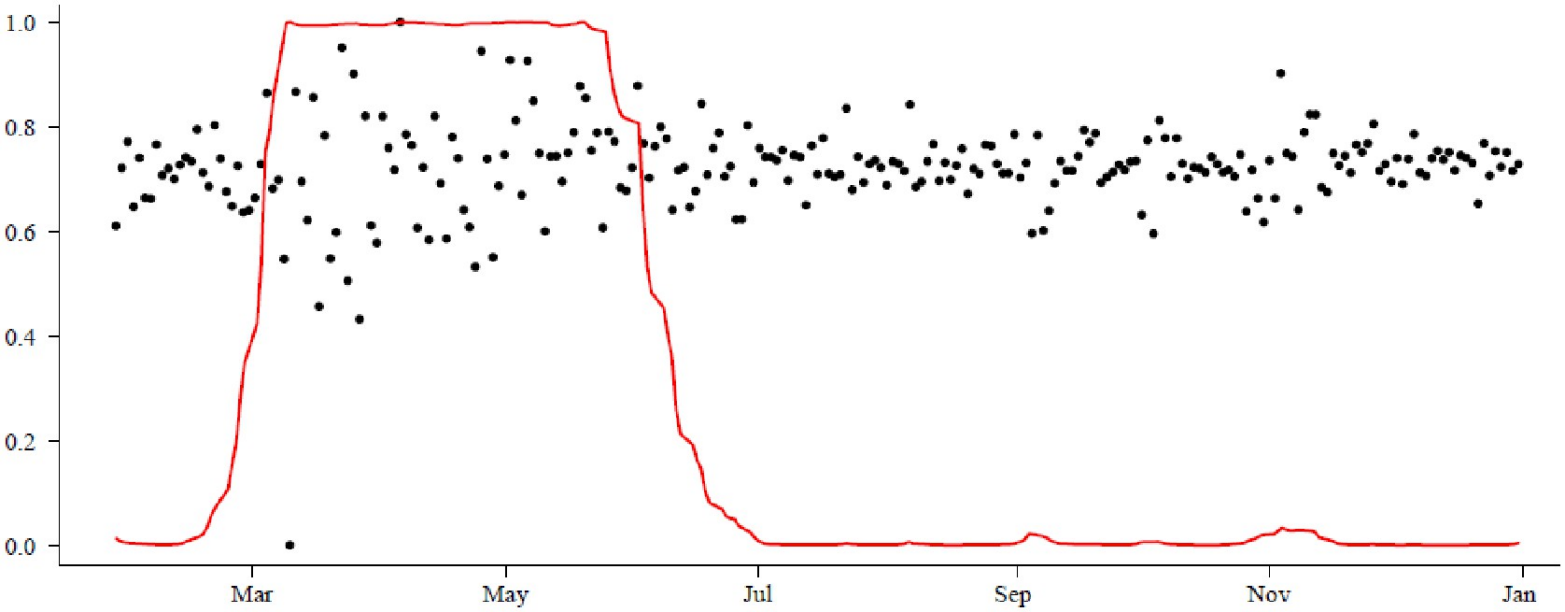
The smoothed probabilities for the two regimes of the crude oil returns confirm two regimes.

As shown in Fig 4, the first regime, covering the period from 27 January 2020 to 30 June 2020, exhibits a period of high volatility, which we have classified as COVID-19 Phase 1. The subsequent stage is characterized by a period of relatively lower volatility, spanning from 1 July 2020 to 31 December 2020, referred to as COVID-19 Phase 2. We conducted a comprehensive analysis of the MS-GARCH models by investigating 18 different combinations that were determined based on the number of regimes. The three GARCH models are the standard GARCH, EGARCH, and GJR-GARCH with the underlying normal (N) or student-*t* (S) distributions.

The analysis shows the presence of two regimes, with each regime following the same heteroskedasticity, but separately. Based on the AIC and BIC selection criterion, we found that the best model is GJR-GARCH(1,1) with an underlying normal (N) distribution for each regime. The parameters are estimated using maximum likelihood estimation (MLE), as shown in Table 2.

**Table 2:** Estimation results of parameters for MS-GARCH in two regimes with the model specification of GJR-GARCH(1,1) with an underlying normal (N) distribution for each regime.

Estimates	Regime 1	Regime 2
*ω*	11.523*	1.486**
	(9.6684)	(0.7199)
*α*	0.009	0.001
	(0.0017)	(0.0016)
*γ*	0.932**	0.323**
	(0.5250)	(0.2087)
*β*	0.386**	0.530***
	(0.2238)	(0.1646)

Note:

*,**,** refers to statistical significance of 1%,5% and 10%, respectively.

Table 3 provides the transition matrix for the two states, with *k* = 1 representing COVID-19 Phase 1 and *k* = 2 representing COVID-19 Phase 2. The probability of remaining in a period of high volatility, i.e., COVID-19 Phase 1, is 0.9805, and the probability of transitioning from COVID-19 Phase 1 to a lower volatility regime, i.e., COVID-19 Phase 2 is 0.0198. Moreover, the probability of transitioning from a high volatility regime to a low volatility regime is 0.0056, and the probability of remaining in a low volatility regime once already in that regime is 0.9940.

**Table 3:** Transition matrix.

Probabilities of transitioning		
	*t* + 1 ∣ *k* = 1	*t* + 1 ∣ *k* = 2
*t* ∣ *k* = 1	0.9805	0.0198
*t* ∣ *k* = 2	0.0056	0.9940

**Fig 5:**
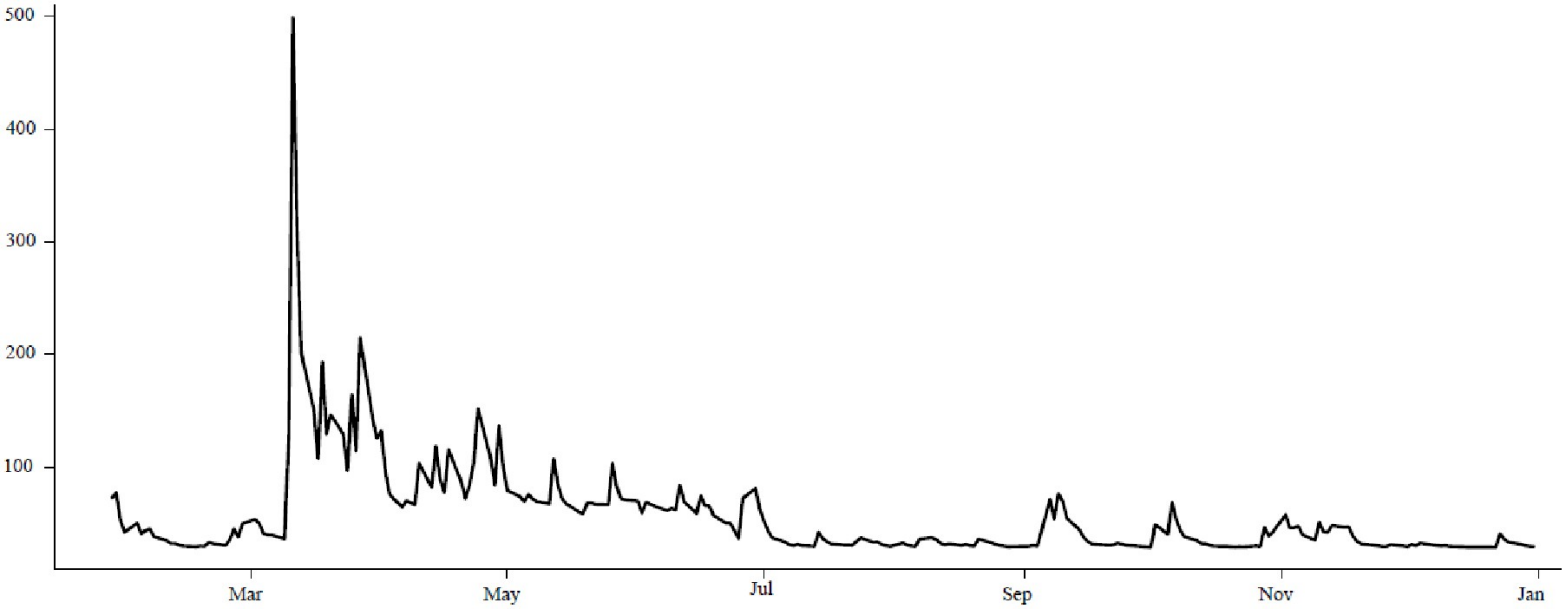
Unconditional volatility of the crude oil prices.

Fig 5 presents the unconditional volatility of the crude oil prices. The stable probabilities (unconditional probabilities) of being in a specific regime, i.e., *i* = 1, 2 are presented in Table 4. The stable probability of crude oil prices remaining in a high volatility regime is relatively less persistent, i.e., approximately 0.22, in contrast to the low volatility regime, which exhibits a persistence of about 0.78.

**Table 4:** Stable probabilities.

Regime 1	Regime 2
0.22	0.78

These findings provide evidence that there was a regime shift in crude oil prices within the first year of the COVID-19 pandemic.

### Macroeconomic variables and PCA

We investigated whether other macroeconomic variables demonstrated significant shifts during these two phases of the COVID-19 period. To achieve this, we performed both the mean equivalence *t*-test and *F*-test for equality of variances on each variable in every phase. The findings from these tests are presented in Table 5.

**Table 5:** The results of *t*-test and *F*-test for the macroeconomic variables.

Mean				Variance		
Macroeconomic variables	COVID-19 Phase 1	COVID-19 Phase 2	2-tailed *t*-test	COVID-19 Phase 1	COVID-19 Phase 2	*F*-test
CCI	147.8	160.5	-6.09	439.8	57.1	7.7
DSI	1231.0	1165.4	20.89	626.4	562.8	1.1
GEI	6195.7	7231.5	-15.6	360542.5	153876.3	2.3
Gold	106.4	144.6	-24.8	220.9	52.9	4.17
OVX	94.1	43.9	9.53	3047.3	52.5	58.1
SP1200	2339.6	2695.8	-14.57	50618.7	19203.5	2.6
WGBI	1020.3	1072.2	-28.2	233.1	170.8	1.3

The *t*-tests show absolute values exceeding 2, indicating a statistically significant and necessitating the rejection of the null hypothesis of no difference in means between the two phases. Hence, the results indicate a statistically significant difference in the means of the variables between the two phases of the COVID-19 pandemic. Similarly, the standard deviations, which indicate volatility, exhibit statistically significant *F*-test for both phases of COVID-19. This leads to the rejection of the null hypothesis, confirming a significant difference in shock levels during both phases.

Fig 2 on the standardized macroeconomic variables that was presented earlier shows signs of multicollinearity between variables arising from significant correlation. Therefore, PCA is performed to optimize feature extraction and reduce the number of independent macroeconomic variables included in the analysis. The two most prominent components that have been identified can be used as indicative of overall market sentiment at the macro level.

**Fig 6:**
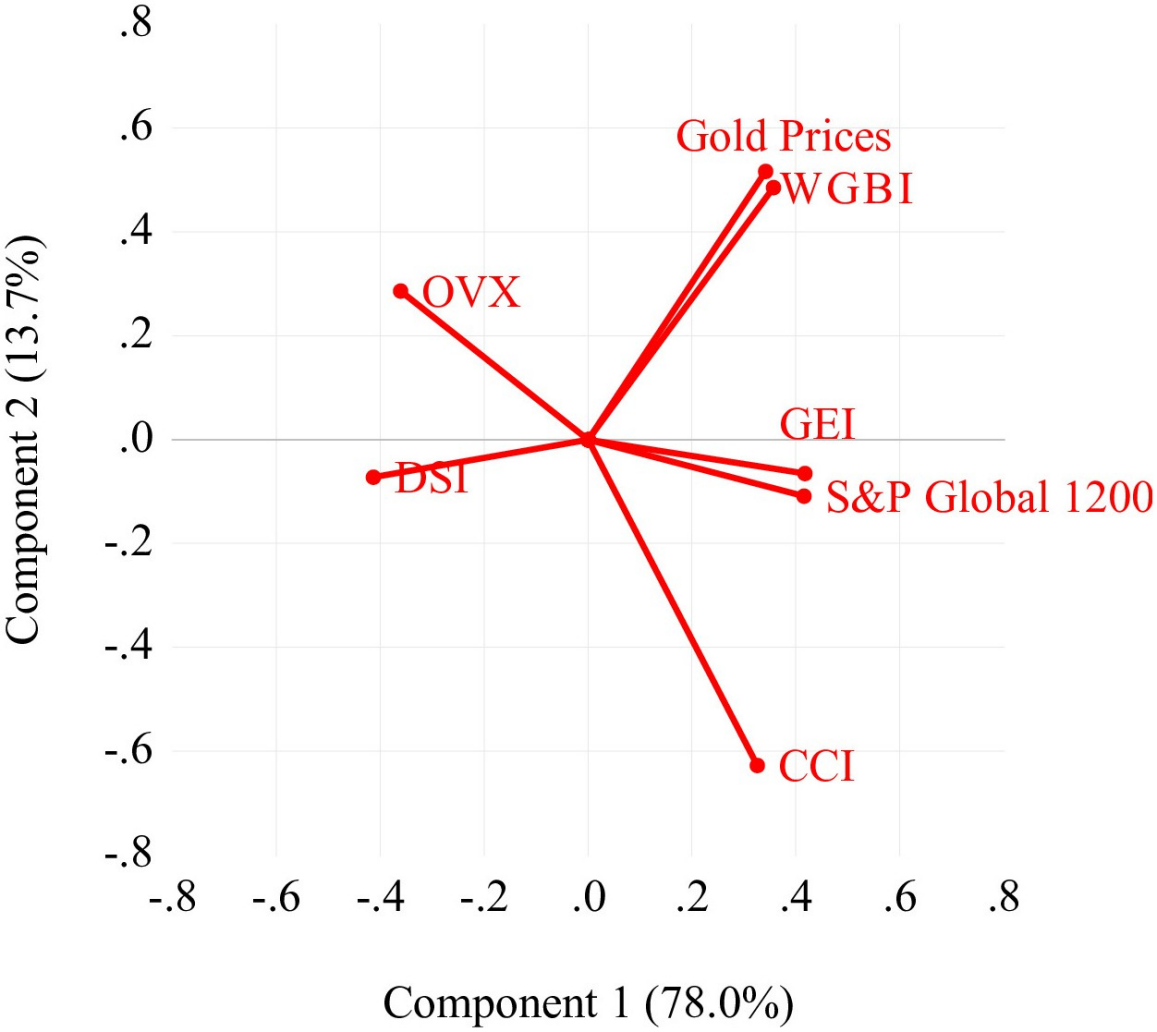
The orthonormal loadings of the seven macroeconomic variables.

**Fig 7:**
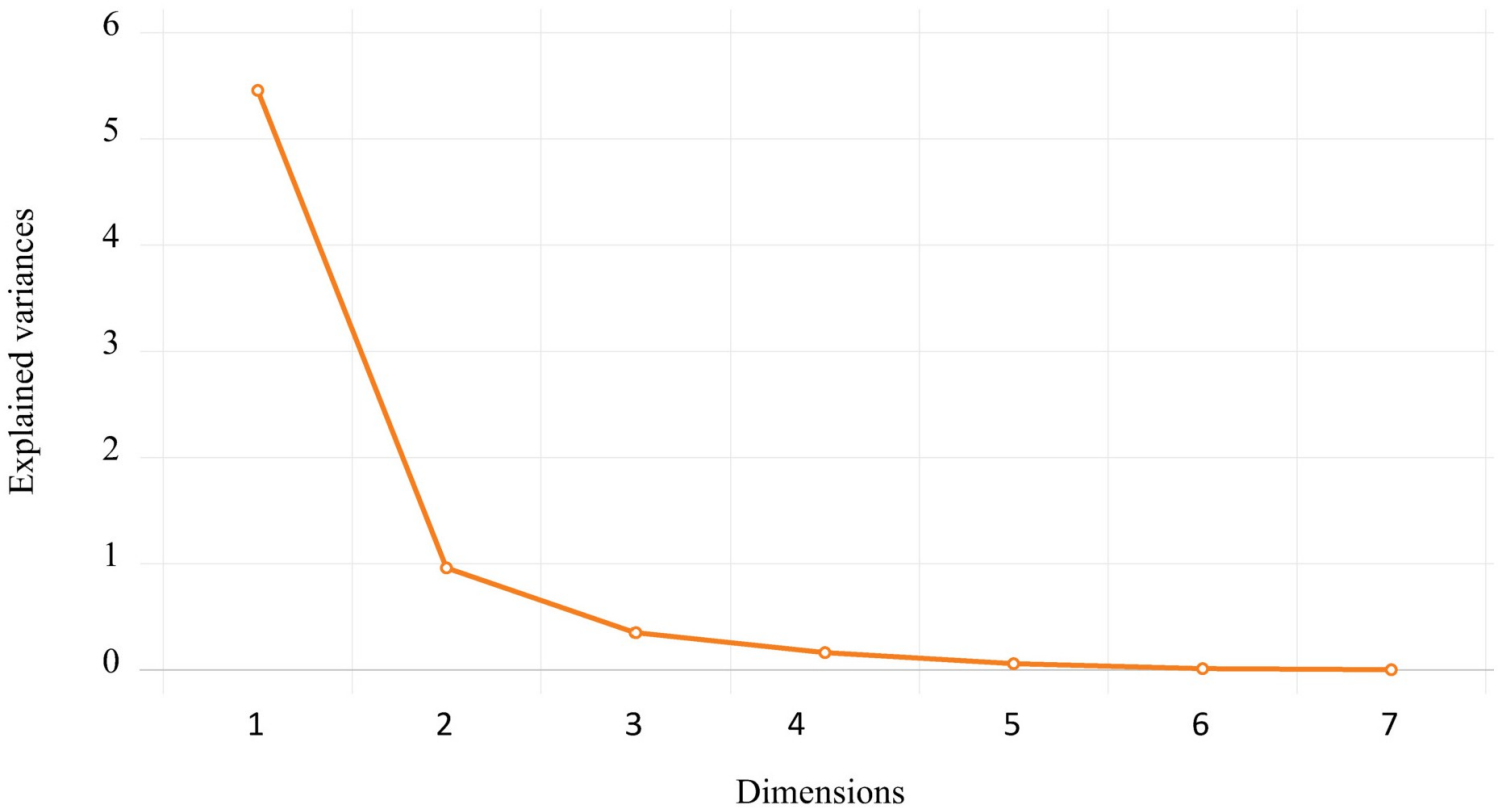
The scree plot from the principal component analysis of the macroeconomic variables.

Fig 6 shows the orthonormal loadings of the seven macroeconomic variables in this study. The scree plot in Fig 7 illustrates that the first principal component accounts for approximately 78% of the variance, while the second principal component contributes 13.7%. Given that the contribution of the remaining components is below 9%, the initial selection for the regression analysis will consist of the first two components, which serve as explanatory variables.

Based on the reduced components, we identified the two most influential principal factors, namely the systematic risk (*SR*) and the economic interdependence factor (*EIF*).

As illustrated in Table 6, *SR* has an almost identical absolute value of approximately 0.35 across all factor loadings. This finding suggests that *SR* impacts all macroeconomic variables, which is why it has been named accordingly. *SR* is associated with the entire market or the whole market segment, influencing the broader economy as a whole.

**Table 6:** Principal factors loadings for macroeconomic variables.

Economic risk indicators	*SR*	*EIF*
CCI	0.326583	-0.627709
DSI	-0.413669	-0.072911
GEI	0.417612	-0.065655
Gold Prices	0.342602	0.516855
OVX	-0.360483	0.285945
SP Global 1200	0.415586	-0.108885
WGBI	0.357472	0.485381

Likewise, as crude oil prices have a substantial effect on all commodities, the results demonstrate that the factor loadings for both the CCI-Core Commodity Index and gold prices were the highest for the second factor. This factor is therefore identified as *EIF*.

**Fig 8:**
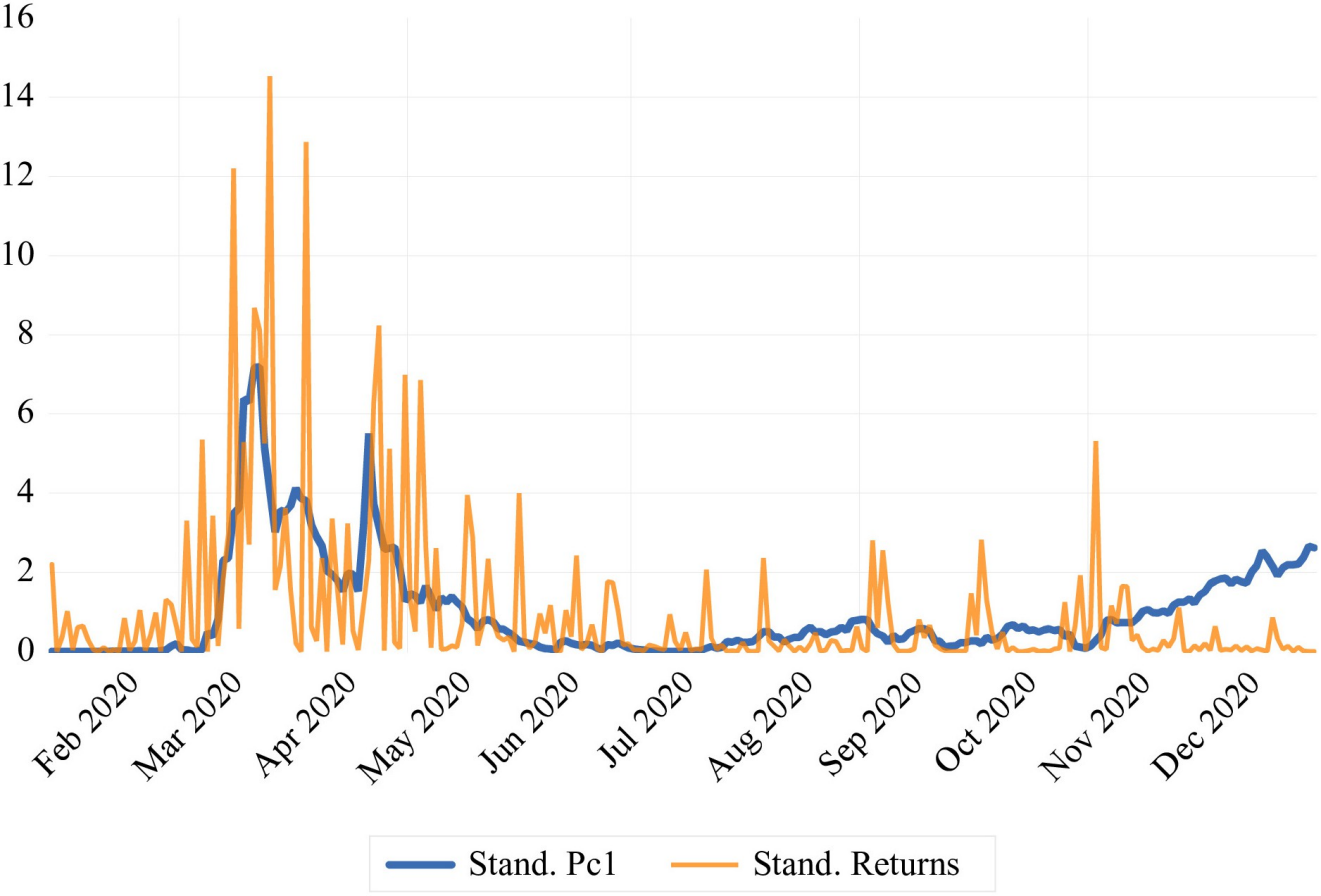
Co-movement of crude price oil prices’ standardized squared returns with the first principal component factor.

**Fig 9:**
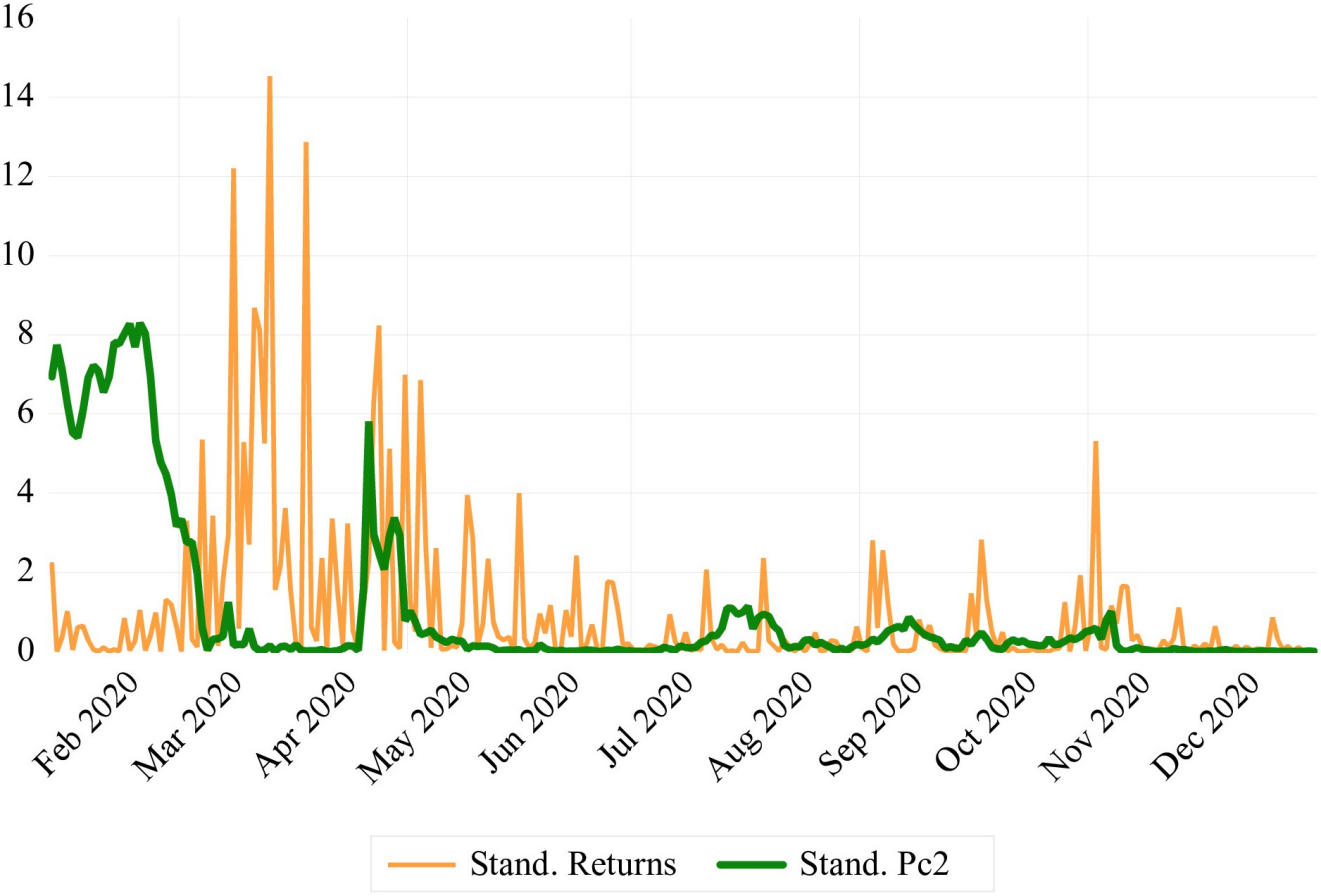
Co-movement of crude price oil prices’ standardized squared returns with the second principal component factor.

In order to support the inclusion of these two components in our eventual regression analysis, we have plotted the co-movement of these two factors with crude oil prices’ standardized squared returns. Fig 8 illustrates that the *SR* displays high volatility during the first stage of the COVID-19 pandemic, in line with the movements in crude oil prices. Similarly, the *EIF* depicts exactly an inverse relationship with the fluctuations in crude oil prices as shown Fig 9. The observed co-movement supports our PCA findings, indicating that these factors accurately reflect market sentiments related to the volatility of the crude oil market.

### Risk contagion

The study further explored the risk contagion between the death and recovery rates associated with COVID-19, and the crude oil prices. For this purpose, we employed the multivariate BEKK-GARCH model. Our analysis reveals the presence of asymmetry, leading us to implement the extension of multivariate BEKK-GARCH with asymmetry. The results are presented in Table 7.

**Table 7:** Estimation results of parameters for multivariate BEKK-GARCH model.

Parameters	Parameter estimate
*c*_11_	0.555*
*a*_11_	-0.119*
*a*_21_	0.009**
*a*_31_	-0.0127
*b*_11_	0.826*
*b*_21_	-0.008*
*b*_31_	0.0275*
*d*_11_	0.697*
*d*_21_	-0.010***
*d*_31_	0.018

Note:

*, **, and *** refer to the statistical significance of 1,5 and 10%, respectively.

In the multivariate BEKK-GARCH model, the diagonal arch effect terms *a*_*ii*_ represent the short-run persistence of the variable’s own past innovations on itself. The diagonal elements of *b*_*ii*_ reflect long-run persistence, whereas *a*_*ij*_ and *b*_*ij*_ represent the cross-market risk contagion effects among selected commodities in the short-run and long-run, respectively.

The results indicate that *a*_11_ = −0.1193 and *b*_11_ = 0.8262 are statistically significant, signifying the impact of crude oil price shocks on short-run volatility. The high value of *b*_11_ provides strong evidence of the substantial impact of past conditional variance on long-term volatility persistence in crude oil prices. Thus, current volatility has implications for future volatility in the long run.

The significance value of *a*_21_ = 0.0091 indicates that there was a spillover effect from the past death rate on the crude oil prices in the short run. However, the non-significant value of *a*_31_ = −0.0127 implies that the recovery rate did not have an immediate impact on the crude oil prices.

The GARCH parameters exhibit statistical significance, indicating a long-term persistence across different types of innovations. Specifically, the values of *b*_21_ = −0.008 and *b*_31_ = 0.0275 suggest that the long-term spillover effect is predominantly driven by the recovery rate rather than the death rate. This implies that the recovery rate has a stronger influence on the persistence of the spillover effect over time.

Moreover, the results demonstrate a significant diagonal factor *d*_11_ for the asymmetry, implying an asymmetric effect on the volatility process of crude oil prices. Also, the significance of *d*_21_ implies the measurable asymmetric impact of death news on the oil prices, whereas there was no significant asymmetry induced by the number of recoveries.

### Regression equation and parameters

We have now investigated the impact of the COVID-19 indicators on the risk associated with crude oil prices, while also considering the influence of macroeconomic variables. Table 8 shows the estimate of the regression parameters.

**Table 8:** The estimate of the regression parameters.

Variables	*COPR*_*T*_		
	Full Sample	COVID-19 Phase 1	COVID-19 Phase 2
*SR*_*t*_	0.8133 (0.0308)	0.5116 (0.0539)	0.2956 (0.0339)
*EIF*_*t*_	-0.1104 (0.0505)	-0.6379 (0.0991)	0.2372 (0.0797)
*Death*_*t*_	0.9115 (0.0680)	0.9357 (0.1186)	0.2117 (0.0469)
*Recovery*_*t*_	-0.26265 (0.0952)	-1.9170 (0.3161)	0.0972 (0.0482)
*Constant*_*t*_	3.4306 (0.0363)	2.8819 (0.2018)	2.6815 (0.0610)
No. of observations	244	112	132
*Adj*.*R*^2^	0.9126	0.9332	0.4031

Throughout the entire sample period and across individual phases, i.e., COVID-19 Phases 1 and 2, it is evident that the point estimate for the *SR*_*t*_ remains positive. This suggests that the risk associated with the crude oil market was consistently moving in the same direction as other macroeconomic variables in all phases. However, in the full sample, both *EIF*_*t*_ and *Recovery*_*t*_ exhibited a negative association with the risk. Notably, the highest point estimate was observed for *Death*_*t*_, indicating that the risk in crude oil prices was significantly influenced by the number of COVID-19 deaths.

The results show that during COVID-19 Phase 1, it was evident that the point estimate with the largest absolute value was no longer associated with *Death*_*t*_, but rather with *Recovery*_*t*_. Hence, the favorable news regarding the recovery had a more significant inverse impact on risk during the high volatility of COVID-19 Phase 1, in comparison to the full sample.

COVID-19 Phase 2 exhibits almost identical and positive associations with *SR*_*t*_, *EIF*_*t*_ and *Death*_*t*_, and a weak association with *Recovery*_*t*_. The reason behind this can be attributed to the proactive implementation of precautionary measures and strict enforcement of lockdowns during this period, resulting in a reduced level of volatility in the crude oil price market.

### Section 5: Conclusion

The COVID-19 pandemic significantly affected not only the healthcare system but also various industries around the world, including the financial industry. As crude oil prices experienced greater volatility, they generated increased uncertainty, thereby causing economic instability for countries that depended on the import and export of oil.

This paper provides a structured analysis of how COVID-19 indicators influenced the transmission of risk to crude oil prices. In particular, we investigated the impact of COVID-19 news, represented by daily global death and recovery rates, on the volatility in crude oil prices.

Through the implementation of the multivariate BEKK-GARCH model, it was determined that the short-term volatility observed in crude oil prices was a result of its past innovations and the COVID-19 death rate. On the other hand, the long-term volatility was impacted by the spillover effects stemming from the news coverage related to COVID-19. Specifically, our analysis demonstrates a significant and positive impact of the COVID-19 death rate, regarded as unfavorable news, on the volatility of crude oil prices. Conversely, the volatility in the COVID-19 recovery rate, which signifies positive news, is negatively correlated with the volatility of crude oil prices. Furthermore, the results of our study reveal an asymmetric impact, showing that unfavorable news has a greater effect on crude oil prices in comparison to favorable news.

The results of our study offer insightful information about how oil prices are volatile during pandemics, thereby offering useful recommendations for investors, portfolio managers and policymakers.

For investors and portfolio managers, the implications derived from these results indicate that the pandemic has highlighted the necessity of rigorously analyzing the impact of shocks on crude oil volatility. Additionally, it is crucial to take into account global macroeconomic factors when formulating investment strategies. By closely monitoring and analyzing these indicators, investors and portfolio managers can obtain valuable insights into the relationships among various risks, thus improving their ability to predict and manage volatility in the market more effectively. For example, during periods of increased uncertainty, investors and portfolio managers can hedge the risks of their investments by increasing their holdings in stocks that are not heavily influenced by volatility in oil prices.

The policymakers, alongside financial regulators, should strengthen their financial markets by adopting effective financial policies and establishing a comprehensive institutional framework that ensures resilience against external shocks.

The results of our study may also provide valuable insights for policymakers to formulate policies that effectively reduce uncertainty in the global oil markets. These insights emphasize the need for an in-depth understanding of various renewable energy sources and their influence on oil prices. A study by [38] indicates that renewable energies such as hydroelectric, geothermal, wind, and biomass may contribute significantly to mitigating the economic impacts of oil price volatility.

## Supporting information

S1 Table Macroeconomic variables indexes. The details about the macroeconomic variables indexes used in the paper.
(ZIP)
