# Supplementary material for: Risk contagion of COVID-19 to oil prices: A Markov switching GARCH and PCA approach
Source: PLoS One. 2024 Dec 5;19(12):e0312718. doi: 10.1371/journal.pone.0312718 (PMC11620598; doi:10.1371/journal.pone.0312718)
Supplement: S1 Table — The details about the macroeconomic variables indexes used in the paper. (ZIP) [file pone.0312718.s001.zip › S1_Table.pdf]

Risk contagion of COVID-19 to oil prices: a Markov switching GARCH and PCA approach

Supporting information

S1 Table. Macroeconomic Variables Indexes. The details about the macroeconomic variables indexes used in the paper.

S1 Table

Index Name	Details
CBOE Crude OIL ETF Volatility Index	The Cboe Crude Oil ETF Volatility IndexSM (OVX) is an estimate of the expected 30-day volatility of crude oil as priced by the United States Oil Fund (USO). Like the Cboe VIX Index, OVX is calculated by interpolating between two time-weighted sums of option mid-quote values - in this case, options on the USO ETF. The two sums essentially represent the expected variance of the price of crude oil up to two option expiration dates that bracket a 30-day period of time. OVX is obtained by annualizing the interpolated value, taking its square root and expressing the result in percentage points.
MSCI World index	The MSCI World Index is a broad global equity index that represents large and mid-cap equity performance across all 23 developed markets countries. It covers approximately 85% of the free float-adjusted market capitalization in each country.
FTSE World Government Bond Index(FTSE WGBI)	A broad index providing exposure to the global sovereign fixed income market, the index measures the performance of fixed-rate, local currency, investment-grade sovereign bonds. It comprises sovereign debt from over 20 countries, denominated in a variety of currencies
CoreCommodity Index(CCI)	A basket of 19 commodities, including agriculture, precious metals, and industrial metals, the Index acts as a representative indicator of commodity markets
S & P Global 1200 Index	The S& P Global 1200 Index is a free-float weighted stock market index of global equities from Standard & Poor's. The index was launched on Sep 30, 1999 and covers 31 countries and approximately 70 percent of global stock market capitalization.
Daily Gold Prices	These are daily XAU spot gold prices. These are the daily closing prices which are recorded.

Table S1 continued from previous page

The Bloomberg Dollar Spot Index (BBDXY)	The Bloomberg Dollar Spot Index (BBDXY) tracks the performance of a basket of 10 global currencies against the U.S. dollar. Its composition is updated annually and represents a diverse set of currencies that are important from a global trade and liquidity perspective.
